# Supplementary material for: Mutations in PCYT2 disrupt etherlipid biosynthesis and cause a complex hereditary spastic paraplegia
Source: Brain. 2019 Oct 22;142(11):3382–97. doi: 10.1093/brain/awz291 (PMC6821184; doi:10.1093/brain/awz291)
Supplement: awz291_Supplementary_Data [file awz291_supplementary_data.zip › awz291-Suppl_data/Supplementary_Data4.zip!/Supplementary information 4 - plasma lipidomics.zip!/Plasma lipidomics/Control vs Patient3b/Statistical plots and data/LPE[O] species/Random forest.pdf]

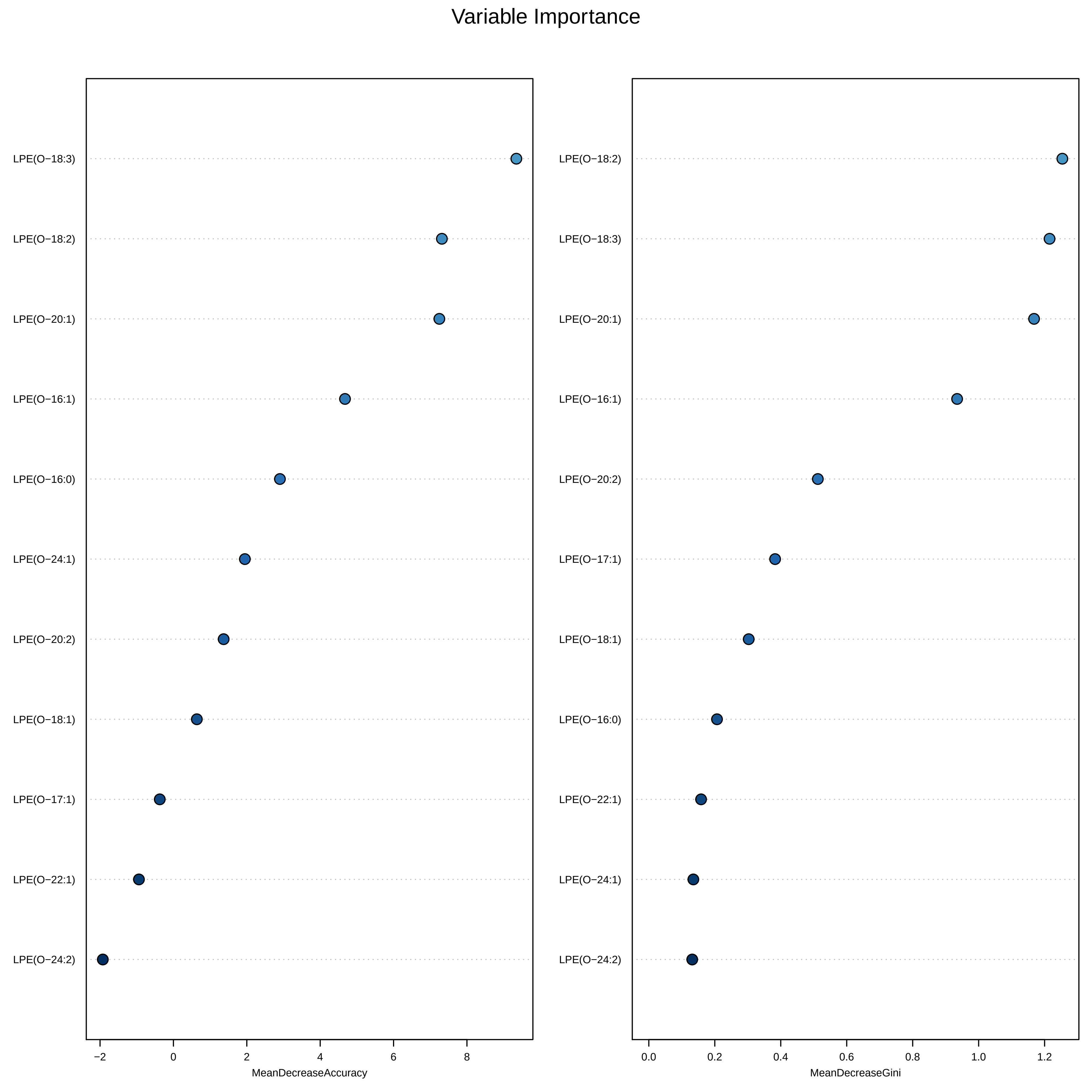

Variable Importance
LPE(O−18:3)
LPE(O−18:2)
LPE(O−20:1)
LPE(O−16:1)
LPE(O−16:0)
LPE(O−24:1)
LPE(O−20:2)
LPE(O−18:1)
LPE(O−17:1)
LPE(O−22:1)
LPE(O−24:2)
−2
0
2
4
6
8
MeanDecreaseAccuracy
LPE(O−18:2)
LPE(O−18:3)
LPE(O−20:1)
LPE(O−16:1)
LPE(O−20:2)
LPE(O−17:1)
LPE(O−18:1)
LPE(O−16:0)
LPE(O−22:1)
LPE(O−24:1)
LPE(O−24:2)
0.0
0.2
0.4
0.6
0.8
1.0
1.2
MeanDecreaseGini
